# Etiology, Diagnosis, Complications, and Management of Acute Otitis Media in Children

**DOI:** 10.7759/cureus.28019

**Published:** 2022-08-15

**Authors:** Abdullah Jamal, Abdulla Alsabea, Mohammad Tarakmeh, Ali Safar

**Affiliations:** 1 Department of Otolaryngology, Zain Hospital, Kuwait, KWT; 2 Department of Cardiology, Mohammed Bin Khalifa Bin Salman Al Khalifa Specialist Cardiac Centre, Awali, BHR; 3 Department of Otolaryngology, Al-Adan Hospital, Kuwait, KWT

**Keywords:** streptococcus pneumonia, myringotomy, prophylactic antibiotics, anesthesia, acute otitis media

## Abstract

Acute otitis media (AOM) is the most common infectious disease encountered by children under the age of two years and the most common cause of antibiotic use in children in the United States. AOM causes irritability, sleeplessness, decreased appetite, imbalance, and dizziness in patients, especially young children. This assessment was conducted to measure the effectiveness of surgical interventions in treating AOM. We reviewed the present findings regarding the etiology, clinical presentations, diagnosis, treatment, and surgical treatment of complications of AOM. Pain associated with AOM (otalgia) can be severe enough to cause parents to seek treatment for their infants or children. Various suggested measures have been used to treat AOM; antibiotic treatment with amoxicillin is still the treatment of choice for AOM, yet other antibiotics may be used in cases of allergy to penicillin or recent use of amoxicillin. Surgical intervention has been introduced and studied as a diagnostic, therapeutic, and preventive measure for AOM; nevertheless, a few studies have shown that surgical interventions are beneficial in treating and preventing AOM compared to the common practice of using antibiotics. Overdiagnosis of AOM is widespread, leading to injudicious antibiotic use, which contributes to antibiotic resistance. Further management should be determined together with the parent, particularly if observation is the primary intervention.

## Introduction and background

Acute otitis media (AOM) is defined as "the rapid onset of signs and symptoms of inflammation in the middle ear" [1]. Recurrent otitis media occurs when episodes of AOM are repeated on three separate and well-documented occasions in a period of the last six months or four or more occasions in the last 12 months [2]. A more complicated presentation of otitis media is otitis media with effusion (OME), which is considered chronic otitis media. Chronic otitis media presents with liquid in the middle ear cavity and without the signs and symptoms of acute otitis media [1]. Tympanostomy tube insertion and myringotomy are the most frequently implemented surgical interventions [3].

AOM is the most common diagnosis worldwide. The Centers for Disease Control and Prevention (CDC) surveillance data showed that the prevalence of AOM in the United States is increasing (150%). Before the age of two years, 70% of children will have encountered at least one AOM episode. Studies have shown that AOM is the main cause of empiric antibiotic prescription in the United States. Various types of otitis media have been identified depending on the presentation of symptoms and complications.

Otolaryngology is a branch of medicine that deals with the ear, nose, and throat from anatomical, physiological, and pathophysiological aspects to assist in the treatment of AOM, and a thorough understanding of these aspects should be assured [4].

In this study, we reviewed the current findings regarding the etiology, clinical presentations, diagnosis, treatment, and surgical treatment of complications of AOM.

## Review

Anatomy of the ear

The ear is a sensory organ that consists of the outer, middle, and inner ears. The outer ear is responsible for collecting and admitting sound waves. It consists of the auricle and meatus, which are also known as the external tubular auditory canal that ends at the ear and drum (outer side of the tympanic membrane). The inner side of the tympanic membrane forms a cavity within the temporal bone, which is referred to as the middle ear. Auditory ossicles are located in the middle ear, malleus, incus, and stapes. The eustachian tube (ET) is a tube that links the back of the nose to the middle ear, whereas the inner ear is responsible for maintaining balance with the aid of the semicircular canals and vestibule, and conducting hearing through the cochlea [5].

Acute otitis externa

Acute otitis externa (AOE), also known as swimmer’s ear, is an acute inflammatory reaction that affects the subdermis of the external auditory canal and is caused by bacterial infection. During summer, as people tend to swim and partake more in water sports, the prevalence of AOE increases. Factors such as humidity, warmth, alkaline pH in the external canal, and ear scratching can cause acute otitis externa [6,7]. Pseudomonas aeruginosa and Staphylococcus aureus are the predominant bacterial species isolated from patients with AOE, while other bacterial species are, to a lesser extent, encountered and isolated, namely Proteus mirabilis and Klebsiella pneumonia [8].

Acute otitis media

Acute otitis media (AOM) is purulent or suppurative otitis media. As its name indicates, AOM is characterized by fluid in the middle ear region. This infection of the middle ear cavity is associated with signs and symptoms of rapidly emerging acute inflammation. Ear pain (otalgia) and fever are the most common signs seen in AOM; these distressing signs and their cause are the most common reasons for children’s visits to the clinic [9]. The connection between the middle ear, which is air-filled and coated with respiratory mucosa, and the nasopharynx is through the ET, which controls fluid drainage and pressure in the middle ear. The anatomy of the ET is slightly different in infants and children than that in adults; the shorter, steeper angled tube found in infants and children transforms to its adult version when a child reaches the age of seven. The tube is usually closed at rest. Sporadic jaw movement due to yawning and swallowing opens it [10]. Any anatomical or physiological dysfunction in the ET may cause AOM [11]. The pathophysiology of AOM is simply due to inflammation in the ET, which prevents fluid drainage from the middle ear cavity, and this fluid retention eventually turns into purulent effusion, which is characteristic of AOM. The middle ear cavity is normally a sterile site. The presence of viral or bacterial pathogens, and that, alongside allergic reactions, is usually the initiation factor for ET inflammation that will eventually develop into a sequela of AOM [9]. Many risk factors have been associated with recurrent AOM, including male sex and genetic susceptibility. For instanceNative Americans have a very high prevalence of AOM compared to African Americans; additionally, daycare attendance usually increases the chances of infection transmission. Other factors, such as pacifier use, which exacerbates ET dysfunction due to sucking pressure, are also associated with recurrent AOM, as are lack of breastfeeding and passive smoking [12,13]. The incidence peak of AOM appears to be at the age of 3-18 months [9].

Etiology

Various etiologies have been associated with AOM, such as the biology of the middle ear cleft, differences in anatomical structures between individuals, nasopharynx cell biology, and variations in the immune response to microbial invasions [14]. Viral pathogens, bacterial pathogens, and genetics have all been associated with AOM; however, bacterial pathogens are believed to be the main causative agents of AOM.

Viruses

Various clinical studies have evaluated the role of viral pathogens and viral infections as probable etiologies of AOM. Upper respiratory infections caused by viruses such as the respiratory syncytial virus (RSV), influenza virus, and adenovirus pose a greater risk of AOM than the colonization of the nasopharynx with Streptococcus pneumoniae or Haemophilus influenzae [15]. Additionally, viral upper respiratory tract infections (URTI) cause inflammation of the ET, negative ear pressure, and displacement of secretions in the nasopharynx containing the URTI's causative viral agents into the middle ear cavity [16]. One study showed a significant correlation between AOM and laboratory-documented epidemics of respiratory viruses [17]. Respiratory syncytial virus (RSV) is known for its contribution to bronchiolitis and pneumonia in young patients, and its ability to cause acute respiratory illnesses in almost all age groups. According to the CDC, RSV is a common respiratory virus that usually causes mild cold-like symptoms. Studies have shown that RSV-specific intravenous (IV) immunoglobulin prophylaxis and antiviral treatments are effective in preventing AOM episodes [18].

Bacteria

Isolation of bacterial pathogens from AOM effusion revealed strains of the following bacteria: Streptococcus pneumoniae, Haemophilus influenzae, Moraxella catarrhalis, Streptococcus pyogenes, Staphylococcus aureus, Viridians streptococci, and Pseudomonas aeruginosa. Streptococcus pneumoniae (pneumococcus), a gram-positive, diplococcic, spherical bacterium, was found to be the most common cause of AOM in all age groups. It is responsible for more than 50% of AOM; thus, penicillin-resistant S. pneumoniae is the main cause of treatment failure and recurrence of AOM [19]. The most commonly identified serotypes of S. pneumoniae that are associated with AOM are 19F, 23F, 14, 6B, 6A, 19A, and 9V. After S. pneumoniae, non-typable H. influenzae is responsible for approximately 20% of AOM episodes in children aged <6 years, around 50% of which produce ꞵ-lactamases [20,21]. Moraxella catarrhalis has a prevalence rate of approximately 10-15%; however, most of its strains develop resistance due to ꞵ-lactamase production, making it responsible for AOM recurrence [22]. Group A Streptococcus is common in older children and is mainly isolated in cases of perforated eardrums and mastoiditis [23].

Genetic Factors

Genetic factors are mainly related to defects in the innate immune response of certain individuals, mainly responses linked to TNFA-863A, TNFA-376G, TNFA-238G, IL10-1082 A, and IL6-174G alleles, making them more susceptible to AOM episodes [24]. These responses alter the production of cytokines that facilitate an inflammatory response, leading to more frequent episodes of AOM [25]. Treatment should consider targeting the cytokines responsible for mucin production and mucous secretion, and the genes responsible for their expression [26]. Various vaccines have been studied as a prophylactic measure to reduce the frequency of recurrent AOM, among which the pneumococcal vaccine (PCV7) was found to be effective in preventing otitis media caused by pneumococcal serotypes [27].

Clinical presentation

The main symptom in patients presenting with AOE is pain, which may be severe. To distinguish AOE from AOM, the pinna or tragus of the patient is moved, which aggravates the pain in patients with AOE. Pain develops continuously for up to two days [28]. A small amount of milky white discharge is usually accompanied by edema and redness of the ear canal [29]. AOM mostly affects infants and children, and the inability of children to verbalize their complaints makes it difficult to know exactly what symptoms they are facing. Infants and children with AOM are usually irritable, sleepless, have poor appetites, and frequently tug their ears due to otalgia. Upper respiratory tract infections are the main triggers of AOM; thus, patients may have rhinorrhea. Fluid accumulation in the middle ear canal may cause a decrease in hearing, and excessive fluid retention may cause balance problems or dizziness [30].

Complications of AOM requiring surgical treatment 

Well-diagnosed and promptly treated AOM is not usually associated with serious complications; nevertheless, persistent symptoms and treatment failures may increase the risk of hearing loss. Some complications of AOM require immediate surgical interventions, such as rupture of the tympanic membrane, treatment failure or antibiotic resistance, AOM affecting immunocompromised patients, and severe otalgia that does not respond to treatment, or hearing impairment, which may develop into hearing loss. If the hearing loss is prolonged, it may affect the child’s IQ and speaking ability, or develop into recurrent AOM [31].

Diagnosis

Proper and correct diagnosis is critical as it leads to appropriate management. In the case of AOE, diagnosis is made by physical exam. A thorough history and careful clinical examination are needed to assist in the diagnosis of AOM. A history of rapid onset signs of inflammation with effusion in the middle ear is evidence of AOM infection. Pneumatic otoscopy is required to examine the mobility of the tympanic membrane of a patient. If a large volume is seen with a flat tympanogram, the tympanic membrane is perforated. Identifying the presence of fluid in the middle ear differs from identifying sterility; thus, AOM cannot be confirmed by tympanometry; rather, it is only ruled out in cases of a normal tympanogram. Similarly, audiometry, which shows a decrease in hearing, does not contribute to the diagnosis of AOM nor helps to distinguish AOM from OME; it only indicates the presence of middle ear fluid [32].

Preventive measures 

Many prophylactic measures can be taken to reduce or even avoid AOM [33]. Although the genetic tendency to develop recurrent AOM cannot be avoided, allergic management can ease and decrease the incidence of AOM episodes [34]. Pneumococcal vaccines can be used and have been shown to be effective in decreasing the incidence of AOM caused by certain serotypes covered by this vaccine. However, S. pneumoniae serotypes, which are not covered by the pneumococcal vaccine, showed an increase in AOM incidence [35]. Although the Haemophilus influenzae type B vaccine is available (Hib), this serotype is only minimally associated with AOM episodes; thus, taking the vaccine is of limited use [36]. Furthermore, the efficacy of the trivalent inactivated influenza vaccine (TIV) and the live-attenuated intranasal influenza vaccine (LAIV) in preventing AOM was studied, and it was found that TIV and LAIV were 30% to 55% effective, respectively, especially during allergy seasons [37]. Long-term chemotherapy with low doses of antibiotics has been studied and proven beneficial in reducing episodes of recurrent AOM; however, this should be used with great caution because of rapidly emerging antibiotic resistance [38]. In cases of an infirm ET, tympanostomy or myringotomy tubes may be used. These small tubes can be interpolated into the tympanic membrane while preserving air in the ear cavity for a prolonged period of time, preventing fluid accumulation within the middle ear cavity, and maintaining pressure equalization and balance [39]. 

Treatment

Treatment of recurrent AOM is divided into two categories: medical and surgical. Medical therapy is mainly attributed to the use of topical antiseptics and topical and oral antibiotics, and surgical treatment is mainly performed by inserting a tympanostomy tube into the middle ear cavity [40]. Restoring the slightly acidic media of the outer and middle ear cavities helps resolve ear infections; thus, decreasing the pH by applying acidic solutions is beneficial [41].

Medical Treatment

Available topical treatments for AOE and AOM with tympanostomy include either a single antimicrobial agent (ciprofloxacin, neomycin, ofloxacin, polymyxin B) or a combination of antimicrobial agents with hydrocortisone available as 2-3% ear drop solutions [42]. Because these agents are used topically, they are generally effective even in the case of microbial resistance, and the only treatment failure is due to drug delivery failure when the drug either cannot reach the affected area or its contact time is minimal [43]. Systemic antibiotics are rarely indicated for AOE; they are only used in cases of immunocompromised patients or those with diabetes mellitus. Symptomatic treatment is very important as patients will mainly complain of otalgia; therefore, otalgia, mild to moderate, should be treated first, and for as long as needed with topical or oral analgesics (acetaminophen or NSAIDs), while oral narcotics may be required for severe otalgia [44]. AAP reviewed the treatment of AOM, and the key statements assured the validity of initial observation and symptomatic treatment for pain management without the use of antimicrobial agents due to the rapidly growing resistance caused by antibiotic misuse, which is dependent on age, the severity of the patient’s case, and ability to access healthcare facilities. If the patient is a child younger than a month, he/she should be treated with antibiotics immediately after the observation period. Cases of moderate-to-severe AOM typically present with severe otalgia and fever exceeding 39°C, which should be treated both symptomatically and with an antimicrobial agent. The use of nasal and oral decongestants, corticosteroids, and antihistamines has shown prolongation of middle ear effusions [45]. None showed minimization or improvement of healing or complications of AOM [46]. Amoxicillin is considered the first-line treatment for the management of AOM, and amoxicillin with clavulanic acid is encouraged to broaden antimicrobial coverage for patients with recurrent AOM and recent history of amoxicillin use in the previous month. A dosage of 80 to 90 mg per kg per day is given orally in two divided doses for 10 days or 90:6.4 mg of amoxicillin clavulanate per kg per day is given orally in two divided doses. Cephalosporin is used for patients with penicillin allergies, and macrolides are used if the patient is allergic to penicillin and cephalosporin. If symptoms persist after two to three days of proper antibiotic administration, diagnosis reassessment is considered, and a confirmed diagnosis without symptom improvement suggests treatment failure, which is first aided by an empiric broadening of antibiotic coverage; further treatment failure requires antibiotic-susceptibility as determined by middle ear fluid cultures obtained by tympanocentesis.

Surgical Treatment

The tympanocentesis procedure involves inserting a tiny needle in the tympanic membrane, extending into the middle ear cavity, and aspirating accumulated fluids, thus reducing middle ear cavity pressure. In addition to being diagnostic, tympanocentesis is therapeutically effective in reducing middle ear pain and pressure due to fluid drainage and improves the delivery of antibiotic agents to the infected middle ear cavity. However, it has not shown any positive effect in shortening the duration of effusion or frequency of AOM recurrence [47]. Recurrent AOM is an indication for tympanostomy tubes, which are used as middle ear pressure-equalizing tubes (PET). Studies have shown the implementation of tympanostomy tubes to significantly reduce mean AOM episodes (1.5x) in a six-month follow-up period [48,49]. Another study compared patients with recurrent AOM taking a placebo with those with tympanostomy tubes and followed them for two years; no difference in episode frequency was observed [50]. However, tympanostomy tubes improved disease-specific quality of life measures in patients [51]. Large improvements in the psychosocial realm of physical suffering, hearing loss, speech impairment, emotional distress, and activity limitations were documented in a multicenter, non-randomized study [52,53]. Tympanostomy tube insertion is a small, easy surgical procedure that requires anesthesia, either general or local, and is not associated with major complications such as sensorineural hearing loss, bleeding due to vascular injury, and interruption of the ossicular chain; some common complications may include ear drainage, either ejection or preservation of the inserted tube, benign inflammation formation, and residual perforation. Myringoplasty is required if perforation is combined with secondary infection and hearing loss. The tympanostomy tube may migrate from its location in the ear canal and move behind the intact tympanic membrane in a rare asymptomatic complication [54]. Performing adenoidectomy alone is not recommended for AOM prevention, and the tympanostomy tube should be considered as it is more beneficial [55]. Chemoprophylaxis with low doses of amoxicillin showed a significant decrease in AOM frequency compared to placebo in two studies with a follow-up period of one year [56]. Surgical intervention should be considered in cases of recurrent AOM, either due to ET dysfunction or bacterial resistance, Down’s Syndrome, and craniofacial abnormalities such as cleft palate [57,58].

Formal examination of hearing and the installation of tympanostomy tubes, either alone or in conjunction with adenoidectomy, are options for continued therapy by an otolaryngologist. According to a Cochrane review, the number of patients without AOM in the included trials was higher among those who had ventilation tubes. There is also evidence that the insertion of ventilation tubes improves the quality of life in the short run. Their usefulness in avoiding recurrent AOM is still being contested, especially considering the difficulties associated with the inclusion criteria of randomized controlled studies [59].

As a result, the clinical practice recommendations of the American Academy of Otolaryngology Head and Neck Surgery currently advocate tympanostomy tubes in children with recurrent AOM and effusion at the time of evaluation [60].

Complications Associated With Surgical Treatment

Tympanocentesis is considered a safe surgical intervention; however, its efficacy in treating AOM is limited, and it is sometimes only considered a diagnostic measure [61]. Myringotomy has an associated complication of barotrauma, which is caused by the use of hyperbaric oxygen treatment [62]. Tympanostomy tube insertion is widely used as a surgical intervention in treating OM, and a set of complications has been associated with this surgery; however, these complications are considered acceptable at their level of incidence and seriousness [63]. One of the most important complications associated with tympanostomy tube insertion is otorrhea, which involves discharges and effusions of the middle ear cavity that drain out to the external ear. This drainage might occur immediately post-operation or a few weeks later [64]. Post-operative otorrhea is classified into simple and chronic; simple otorrhea develops mainly immediately post-operation and lasts for no longer than three weeks, responds to topical treatment, and is not repeated. In contrast, chronic otorrhea is persistent or recurrent drainage that may not respond to topical or systemic treatment [64]. Many children may undergo repeated sessions of tube insertion, and studies have shown that treatment of AOM with tube insertion at a young age (> 2 years) results in a reduced need for repeated tube insertions [65]. Persistent tympanic membrane perforation is mainly caused by tube extrusion, which requires tympanoplasty for closure [66]. Cholesteatomas were reported (1.1%) due to retraction of the tympanostomy tube, with a higher incidence in young children (< 5 years), repeated tube insertions, longer tube incubation periods, chronic otorrhea, and the use of certain tympanostomy tubes (Goode T-tube) [67]. Although surgical interventions are uncommon in cases of AOM, once a patient’s condition requires surgical intervention, it should be evaluated and implemented. Children’s parents should be informed about possible complications of the surgery while emphasizing that surgical intervention is not an ultimate cure for the condition, as patients may develop AOM episodes one-year post-operation [68]. Apart from complications associated with tympanostomy tube insertion, attention is focused on complications associated with the patient’s preparation for the operation, either with general or local anesthesia. Each has its own adverse events that should be evaluated and communicated to the patient and their parents. Common complications of general anesthesia include pain, nausea and vomiting, tooth decay, sore throat, allergy to anesthetic agents, aspiration pneumonia, headache, back pain, and hypothermia. Serious complications include cardiovascular collapse, respiratory depression, nerve injury, pulmonary embolism, idiosyncratic reactions, and death [69]. Local anesthesia has fewer serious side effects such as headache, hypotension, bradycardia, direct nerve damage, hypothermia, urinary retention, and pain, as patients may still feel pain despite local anesthesia [57,58,69].

## Conclusions

Acute otitis media (AOM) is without a doubt one of the most common inflammatory disorders in children. It is a major source of morbidity in children and one of the most prevalent causes of antibiotic prescriptions. However, AOM diagnosis may be problematic because symptoms and indicators are not always conclusive and physical examination can be difficult in this age range. A reddish tympanic membrane alone does not rule out AOM, but a hazy bulging membrane with pneumatic otoscopic characteristics associated with effusion against the backdrop of a typical clinical history is pathognomonic of the condition. Furthermore, AOM management has been highly contested, with various distinct treatment regimens. The adoption of these principles is further confounded by a misunderstanding of the many types of OM. Overdiagnosis of AOM is believed to be widespread, leading to incorrect antibiotic use, which contributes to the development of antibiotic resistance and increases the risk of adverse effects. If observation is chosen as the primary intervention, further management should be determined alongside the parent.
